# UVSegNet: Semantic Boundary-Aware Neural UV Parameterization for Man-Made Objects

**DOI:** 10.3390/jimaging12030092

**Published:** 2026-02-24

**Authors:** Hairun Zhang, Ying Song

**Affiliations:** School of Artificial Intelligence, College of Computer Science and Technology, Zhejiang Sci-Tech University, Xiasha Campus, Hangzhou 310018, China; 2023210602021@mails.zstu.edu.cn

**Keywords:** semantic-aware UV mapping, boundary-sensitive parameterization, neural mapping, UV Unwrapping

## Abstract

UV parameterization is a fundamental step in building textured 3D models, but minimizing texture distortion and ensuring seams are placed along meaningful boundaries remains a challenge. This paper proposes UVSegNet, a novel semantic boundary-aware UV parameterization framework that combines part-level segmentation with geometry-aware parameterization. To address the common seam placement issues in parameterization, we introduce a boundary-aware guided UV mapping module that jointly optimizes geometric accuracy and seam layout. Furthermore, to better handle the cylindrical structures common in man-made objects, we introduce a cylindrical supervision strategy to reduce misalignment and unfolding distortion. Experiments on representative object categories show that UVSegNet outperforms other excellent baseline models in both texture quality and seam quality. Compared to Nuvo, UVSegNet improves the angular distortion (conformality) metric by 24.1% and seam compactness by 60.5% by generating a more compact seam layout. Experimental results demonstrate that UVSegNet outperforms baseline methods in both mapping quality and seam quality, thanks to the complementary mechanism of boundary constraints and geometry-driven modeling.

## 1. Introduction

In the current game and film production workflows, various tools have been developed to support automatic UV unwrapping, but there is still a heavy reliance on manual optimization of UVs to improve texture mapping. Existing automated methods often struggle to balance distortion reduction, seam placement, and texture packing efficiency, forcing technical artists to invest significant effort in cutting, adjusting, and arranging UV maps. This reliance on manual refinement not only limits work efficiency but also impacts texture quality. Therefore, the development of more intelligent and automated UV parameterization methods is highly desirable.

### 1.1. Traditional Surface Parameterization

Traditional surface parameterization maps 3D surfaces to 2D domains while preserving geometric properties such as angles, areas, or lengths. Conformal methods (e.g., LSCM [1] and ABF++ [2]) reduce angular distortion to improve texture mapping quality, whereas stretch-minimizing techniques (e.g., SCP [3] and ARAP [4]) maintain local structural consistency while suppressing stretching. To handle complex geometry, segmentation-based approaches decompose surfaces into multiple charts and optimize seams/unfolding to improve unfoldability (e.g., unified partitioning [5]); for point clouds, free-boundary conformal mapping can further enable high-quality bijective unfoldings (FBCP-PC [6]), supported by theoretical results on bijectivity and distortion control [7]. However, these methods are largely geometry-driven and lack semantic awareness, which can fragment semantically related parts in UV space; they may also suffer from overlap or severe distortion on highly curved or topologically noisy surfaces, often requiring manual boundary refinement.

### 1.2. Neural Surface Parameterization

With the rapid progress of deep learning, neural mapping methods [8,9,10,11,12] have become a powerful paradigm for 3D representation by learning mappings from simple parameter domains (e.g., 2D planes or latent spaces) to complex 3D geometry. AtlasNet [13] is a seminal example, modeling surfaces with a set of learnable 2D patches and establishing the foundation of neural surface parameterization. AtlasNet represents a surface using multiple parametric 2D patches and learns a mapping from each patch to 3D, enabling flexible surface approximation without explicit mesh parameterization. However, as it operates on the whole shape without semantic decomposition, it does not explicitly model component boundaries or seam placement criteria, which may lead to patch boundaries that are not aligned with meaningful structural cues for texturing and editing. This motivates the need for structure-aware guidance when optimizing seams and chart layouts. More recently, PointDreamer [14] adopts a texture-atlas representation with UV mapping in a generative/zero-shot pipeline, highlighting that texture quality depends not only on the UV mapping itself but also on surface sampling and filtering; motivated by this, we improve the sampling strategy to better fit the learning of our framework. To address chart decomposition and cutting [15], learning-based approaches such as OptCuts [16] and AutoCuts [17] propose data-driven cutting strategies, while Nuvo [18] and FlexPara [19] further advance segmentation-based neural parameterization via more general 3D representations—Nuvo performs unsupervised chart segmentation via texture–surface mapping, and FlexPara [19] directly learns UV-friendly cuts by optimizing geometric losses (e.g., conformality and stretching). Despite its strong automation, Nuvo’s [18] chart partitioning is primarily driven by probabilistic atlas assignment and geometric/photometric consistency, and the resulting UV islands may not consistently correspond to true part-level boundaries. In practice, this can cause seams to cut through semantically coherent components or place discontinuities in visually salient regions, increasing the manual workload for artists. Also unsupervised neural parameterization methods [20] attempt to learn global free-boundary mappings, but they are still largely geometry-driven and do not explicitly enforce semantically meaningful seam placement. Overall, both patch-based (e.g., AtlasNet [13]) and segmentation-based neural parameterization (e.g., Nuvo [18]) still lack an explicit mechanism to align seam alignment with object structural/semantic boundaries, which is critical for producing editable and visually coherent texture atlases.

### 1.3. Motivation and Method Overview

In the field of 3D vision and virtual content generation, UV mapping serves as a crucial step for unfolding 3D surfaces into a 2D texture space, where the mapping quality directly affects texture fidelity and visual consistency. In recent years, researchers have combined neural networks with geometric priors to model differential geometric properties such as surface normals, curvature, and geodesic distance, enabling networks to produce geometrically consistent and topologically coherent mappings, thereby effectively reducing seam distortion and structural discontinuities [9,18,21]. However, these approaches primarily focus on optimizing geometric features, while largely overlooking the relationship between UV seams, UV island partitioning, and the actual structural composition of objects. Moreover, since UV island partitioning introduces chart interfaces, achieving globally smooth transitions across chart boundaries is non-trivial and often requires explicit regularization [22]. This limitation often increases the workload for technical artists during the mapping process. For example, Nuvo relies on probabilistic UV island partitioning, which fails to accurately capture true part-level structural boundaries, leading to partial mismatches between seam layouts and the underlying geometric structure. Given the strong correlation between texture mapping and object structure, semantic information can serve as an effective form of structural guidance to improve texture mapping quality and optimize seam placement.

Prior research has demonstrated a strong correlation between UV seam placement and semantic boundaries. Many studies have incorporated semantic labels into the mapping optimization process, employing semantic-aware strategies such as using semantic boundaries to guide texture unfolding and repair, or integrating semantic features into large-scale scene reconstruction to enhance texture consistency [23,24]. While these methods have achieved significant performance at the global scene level, they still lack systematic designs for the fine-grained parameterization of individual objects, particularly in dynamically adjusting seam placement and preserving local geometric details, where the results have been less than optimal.

To acquire semantic information for neural mapping in practical asset creation scenarios, we adopt point cloud semantic segmentation as the primary means of semantic extraction. However, effectively integrating and utilizing semantic information together with geometric precision introduces new challenges. Simply concatenating semantic features with geometric inputs often leads to degraded geometric accuracy and insufficient exploitation of semantic cues. Moreover, many existing point cloud semantic segmentation methods, such as PointNeXt [25], mainly focus on object-level or coarse-grained semantic modeling, making it difficult to produce accurate and stable part-level segmentation within individual objects. This limitation further restricts the effective use of semantic information in local UV parameterization.

To address these issues, we fine-tune a Point-MAE-based segmentation model [26] on the PartNet dataset, enabling robust and detailed part-level semantic segmentation at the point-cloud level across different object categories. The resulting semantic boundaries provide reliable guidance for subsequent seam optimization and geometry-aware UV parameterization. In addition, uniform sampling strategies commonly used for object representation often introduce incompatibilities between the semantic segmentation network and the mapping network, which can degrade texture mapping quality. To preserve geometric fidelity while maintaining semantic consistency, we design a topology-aware point cloud sampling strategy tailored to the overall framework. Furthermore, considering that cylindrical structures are prevalent in many man-made object categories, we introduce a lightweight cylindrical object detection strategy to further ensure geometric accuracy during parameterization.

Our main contribution is the proposal of a novel semantic-guided bidirectional recurrent network architecture for UV parameterization. Using a dihedral angle-based edge detection and adaptive dual-density sampling method, semantic information is obtained through a fine-tuned segmentation network after sampling, while UV parameterization is optimized in an unsupervised manner using a boundary-aware neural formulation to guide seam placement. We also design a multi-loss collaborative supervision module. These designs enable our network framework to effectively capture semantic information, ensuring its guiding role while maintaining the geometric accuracy of texture mapping.

## 2. Method

**Figure 1:**
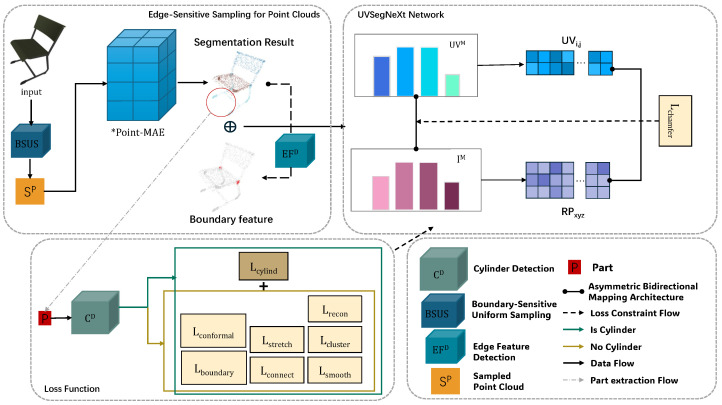
Overall framework of UVSegNet. *Point-MAE denotes the fine-tuned semantic segmentation model used in our framework.

Our proposed framework (Figure 1) addresses the challenges of UV mapping through semantic-boundary-aware and geometry-aware techniques using three coordinated components. Since point cloud-based semantic understanding benefits from global context modeling, while UV parameterization is highly sensitive to local boundary and structural features, we introduce a topology-aware, edge-sensitive adaptive sampling strategy that dynamically adjusts point density based on geometric feature analysis.

The pipeline first converts the input meshes to feature-enhanced point clouds via our adaptive sampling (Section 2.1), then processes them through the Bidirectional Mapping Cycle Network (Section 2.2) for neural mapping, and uses our multi-loss collaborative supervision strategy (Section 2.3). Specifically, semantic segmentation is performed on the input point cloud using a fine-tuned Point-MAE [26] model, which predicts part-level semantic labels for each point. Based on the resulting segmentation, independent UVSegNet networks are trained for individual components, where semantic boundary information is explicitly incorporated to guide seam placement and parameterization. The UV coordinates are optimized via geometrically informed loss terms and finally mapped back to mesh vertices through face-based interpolation, enabling high-fidelity UV reconstruction with improved geometric and semantic consistency.

### 2.1. Boundary-Sensitive Uniform Sampling for Point Clouds

Since the UV parameterization process is highly sensitive to object boundary and structural features, it is necessary to incorporate geometric feature analysis and adaptive density adjustment to ensure sufficient sampling of surface feature regions. To this end, we propose a topology-preserving adaptive sampling strategy that is designed to be compatible with a wide range of point cloud-based semantic segmentation networks.

**Figure 2:**
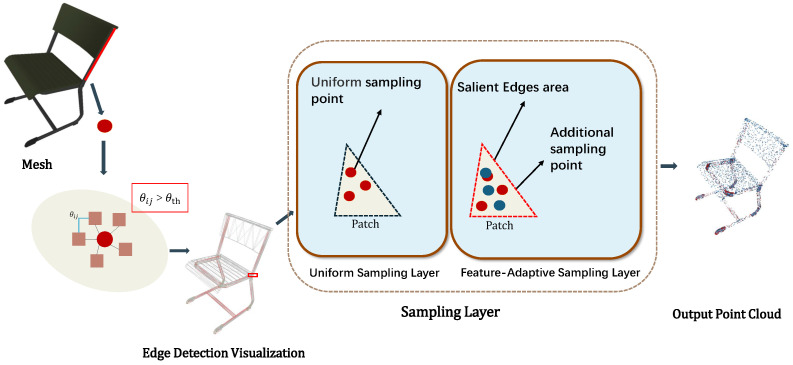
Point cloud sampling pipeline. In the Uniform Sampling Layer, points (red) are uniformly sampled on each face proportional to its area. In the Feature-Adaptive Sampling Layer, additional points (blue) are allocated to salient/edge-related faces to enhance boundary coverage, and a blue-noise distribution is used to prevent local clustering. The final output is the combined set of sampled points.

Specifically, the method analyzes local face connectivity and edge relationships to construct an edge-sensitivity field that characterizes structural discontinuities and boundary regions on the surface.In this field, the sampling density is dynamically adjusted to increase point coverage around geometric feature edges while maintaining overall sampling uniformity in smooth regions. This adaptive sampling strategy provides more informative and boundary-complete point cloud representations for downstream semantic segmentation and UV parameterization. In our method, a fine-tuned Point-MAE model [26] is adopted as a representative semantic segmentation backbone to validate the effectiveness of the proposed framework. The detailed process is illustrated in Figure 2. The input mesh undergoes geometric edge detection by computing the normal vector angles $\theta_{ij}$ between adjacent surface patches, with significant edges identified via the threshold $\theta_{\mathrm{th}}$. The sampling process includes: uniform sampling, which distributes points proportionally to the patch area, and feature-adaptive sampling, which adds extra points in edge patches using blue noise distribution to prevent clustering. The output is a non-uniformly sampled point cloud with boundary-enhanced feature preservation. The specific steps are described in the following subsections.

#### 2.1.1. Edge Detection and Salient Edge Marking

By calculating the dihedral angles and selecting salient edges based on a threshold:

(1) $$E_{\mathrm{sig}}=\left\{\left(i,j\right)\in A|\cos^{-1}\left(n_{i}\cdot n_{j}\right)>\theta_{\mathrm{th}}\right\},$$

where A is the face adjacency matrix, $n_{i}$ and $n_{j}$ are the normal vectors of faces Fi and Fj, respectively, and θth is the predefined threshold. In this work, the threshold is empirically set as $\theta_{\mathrm{th}}=30^{\circ }$. We found the downstream segmentation to be moderately insensitive to the exact value of $\theta_{\mathrm{th}}$ around this setting: using a slightly smaller threshold may introduce spurious edges and lead to over-segmentation, while a larger threshold may weak miss but meaningful boundaries and cause under-segmentation. Therefore, $\theta \mathrm{th}=30^{\circ }$ provides a robust trade-off across categories. The dihedral angle $\theta_{ij}$ is computed by taking the dot product of the normal vectors.

#### 2.1.2. Adaptive Density Allocation and Sampling Implementation

Based on the detected edges, the sampling count is dynamically allocated. Initially, the number of samples per face is assigned proportionally to its area:

(2) $$N_{\mathrm{base}}\left(F_{i}\right)=\left\lceil N_{\mathrm{total}}\cdot \frac{A(F_{i})}{\sum_{j=1}^{\left|F\right|}A\left(F_{j}\right)}\right\rceil ,$$

where $A(F_{i})$ denotes the area of face $F_{i}$, and $N_{\mathrm{total}}$ is the target total number of sampling points. The sampling is implemented using a two-stage hierarchical Uniform Sampling Layer framework. In the first stage, uniform sampling is performed within each face, generating $N_{\mathrm{base}}\left(F_{i}\right)$ points distributed uniformly according to the equation:

(3) $$\begin{array}{cc} p_{k} & =u_{k}v_{0}+v_{k}v_{1}+\left(1-u_{k}-v_{k}\right)v_{2}, \end{array}$$

(4) $$\begin{array}{cc} u_{k} & ∼U\left(0,1\right),\;v_{k}∼U\left(0,1\right),\;u_{k}+v_{k}<1. \end{array}$$

where $\left(u_{k},v_{k}\right)$ satisfy the conditions in Equation (4). The second stage is a Feature-Adaptive Sampling Layer that adds $N\left(F_{i}\right)-N_{\mathrm{base}}\left(F_{i}\right)$ samples on edge faces, employing blue noise distribution [27] to avoid local clustering. This method achieves an effective balance between geometric fidelity and uniformity.

### 2.2. UVSegNet Network

UVSegNet is a neural network framework designed to achieve high-quality UV mapping guided by semantic information. This network adopts a lightweight asymmetric bidirectional recurrent mapping architecture, which reduces the number of parameters while effectively capturing boundary-sensitive features. The network architecture is shown in the UVSegNet network module in Figure 1.

#### 2.2.1. UVMapper with Boundary

To better optimize seam placement in UV parameterization, the proposed network incorporates a boundary-aware attention mechanism that explicitly leverages structural cues derived from semantic segmentation. Given an input point cloud, semantic labels are first predicted using a point cloud-based segmentation network, from which boundary regions between semantic components are identified.

The extracted boundary features are then fused with the corresponding semantic point features and fed into the UV mapping network. By emphasizing points near semantic and geometric boundaries, the boundary-aware attention mechanism guides the network to preserve structural continuity and reduce seam fragmentation during parameterization. In our implementation, a fine-tuned Point-MAE model [26] is adopted as a representative segmentation backbone, while the overall framework remains compatible with alternative semantic segmentation networks.

To extract boundary features, we assume that true boundary points have a significant proportion of neighboring points with different labels. For each point $p_{i}$, we construct a local neighborhood $N_{i}$ using a KDTree with a fixed radius r=0.02. We then compute the ratio of neighbors with labels different from the center point label $l_{i}$:

(5) $$\rho_{i}=\frac{\left|\{l_{j}\neq l_{i}|p_{j}\in N_{i}\}\right|}{|N_{i}|},$$

A point is marked as a boundary point if this ratio exceeds a predefined threshold τ=0.3:

(6) $$I_{\mathrm{boundary}}\left(p_{i}\right)=\left\{\begin{array}{cc} 1 & \mathrm{if}\;\rho_{i}>\tau \\ 0 & \mathrm{otherwise}, \end{array}\right.$$

The value of τ=0.3 follows common practice in point-cloud boundary detection (typically 0.2–0.4) and was empirically fixed at 0.3 based on validation performance.

By concatenating boundary features with geometric coordinates at the channel level, we enable joint learning of geometry and topology, which guides the generation of UV seams. We model the UV mapping as a forward function $F_{uv}:z\to u$, where $F_{uv}$ is implemented using an MLP. Furthermore, to prevent boundary features from dominating the training, we propose a gradient balancing strategy to improve training stability.

#### 2.2.2. Inverse Mapper

For each segmented texture coordinate, the mapping from the 3D point on the surface to the 2D point on the plane should ideally satisfy bijectivity. To achieve this, we design a lightweight inverse mapping module that allows reconstruction at arbitrary scales while enforcing geometric consistency constraints. This module provides backward optimization by propagating gradients through a bidirectional mapping structure to refine the UV mapping process.

Formally, given a predicted UV coordinate $u_{i}\in R^{2}$, we define the inverse mapping function $F_{\mathrm{inv}}:u\to \hat{p}$ modeled by an MLP. The predicted point ${\hat{p}}_{i}\in R^{3}$ should approximate the original input point $p_{i}$:

(7) $${\hat{p}}_{i}=F_{\mathrm{inv}}\left(u_{i}\right)\approx p_{i},$$

To prevent instability and overfitting caused by excessive feedback from the inverse branch, we adopt a gradient clipping strategy during training, ensuring that the inverse mapper does not dominate the learning of the forward UV mapper. This bidirectional training scheme enhances the bijectivity and consistency of the learned UV mapping.

### 2.3. Multi-Loss Collaborative Supervision Module

**Figure 3:**
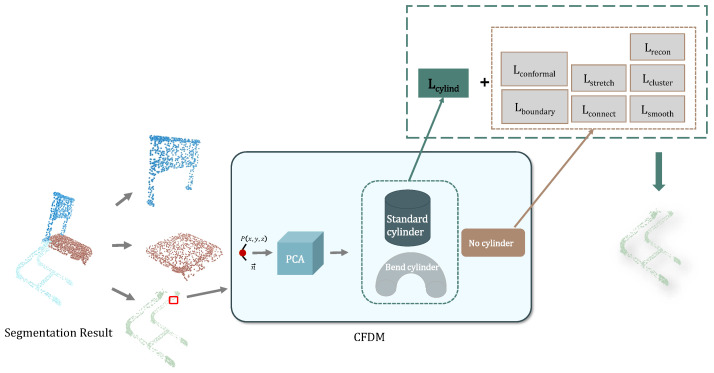
Architecture and workflow of the proposed Multi-Loss Collaborative Supervision Module. collaborative supervision module.

We design a composite loss function composed of multiple sub-loss terms, aiming to simultaneously improve overall geometric reconstruction accuracy, local conformality, boundary compactness, and structural awareness. The specific scheme is shown in the Figure 3.

#### 2.3.1. Structure Awareness of Cylindrical Objects

We observe that certain furniture components, such as chair legs and railings, exhibit pronounced cylindrical structural features. To enhance the network’s geometric modeling capability for such structures, we design a cylindrical loss, which encourages the point sets identified as cylindrical to maintain consistency along one dimension in the UV mapping while exhibiting a periodic variation along the other dimension, thereby preserving the intrinsic cylindrical geometric relationship. Given a set of 3D points $P={\left\{p_{i}=\left(x_{i},y_{i},z_{i}\right)\right\}}_{i=1}^{N}$ and predicted UV coordinates $U={\left\{u_{i}=\left(u_{i},v_{i}\right)\right\}}_{i=1}^{N}$, we define the cylindrical alignment loss as follows: First, extract the *z*-coordinates and the vertical UV coordinates:

(8) $$z=\left[z_{1},z_{2},\ldots ,z_{N}\right],\;v=\left[v_{1},v_{2},\ldots ,v_{N}\right],$$

Normalize both to zero mean and unit variance:

(9) $$\hat{z}=\frac{z-\mu_{z}}{\sigma_{z}+\epsilon },\;\hat{v}=\frac{v-\mu_{v}}{\sigma_{v}+\epsilon },$$

where $\mu_{z}$, $\sigma_{z}$ are the mean and standard deviation of z, and ϵ is a small constant (e.g., $1\times 10^{-6}$ ) to avoid division by zero.

The cylindrical loss $L_{\mathrm{cyl}}$ is then defined as the Mean Squared Error (MSE) between $\hat{z}$ and $\hat{v}$:

(10) $$L_{\mathrm{cyl}}=\frac{1}{N}\sum_{i=1}^{N}{\left({\hat{z}}_{i}-{\hat{v}}_{i}\right)}^{2},$$

This encourages the UV layout to preserve the cylindrical structure along the *z*-axis by mapping it linearly along the *v*-axis.

#### 2.3.2. Reconstruction Loss

To ensure the invertibility between the UV space and the 3D space, this loss measures the reconstruction error of both the forward mapping (uv→xyz) and the inverse mapping (xyz→uv). We jointly train two networks: UVMapperWithBoundary and InverseMapper, and minimize the discrepancy between their predictions and the ground truth:

(11) $$L_{\mathrm{recon}}={∥f_{\mathrm{inv}}\left(U\right)-P∥}^{2}+{∥f_{\mathrm{uv}}\left(P\right)-U∥}^{2},$$

Reconstruction loss $L_{\mathrm{recon}}$ enhances the geometric representation ability of the UV encoding, benefiting both texture back-projection and geometry reconstruction.

#### 2.3.3. Conformal Loss

To preserve angular invariance in texture mapping, we introduce a conformal loss $L_{\mathrm{conformal}}$ that encourages the UV mapping process to retain the original angular structure of each triangular face as much as possible:

(12) $$L_{\mathrm{conformal}}=\frac{1}{\left|F\right|}\sum_{f\in F}{∥{∠}_{f}^{3D}-{∠}_{f}^{UV}∥}^{2},$$

This loss helps prevent severe texture distortion, particularly in regions with complex geometry or high curvature.

#### 2.3.4. Stretch Loss

To maintain local distance ratios, we introduce a stretch loss that penalizes the variation in area of parallelograms spanned by orthogonal tangent vectors during UV mapping. This helps prevent excessive stretching or compression. Given the original edge length $|p_{i}-p_{j}|$ in 3D space and its corresponding edge $|u_{i}-u_{j}|$ in UV space, we compute the squared difference of their length ratios as:

(13) $$L_{\mathrm{stretch}}=\frac{1}{\left|E\right|}\sum_{(i,j)\in E}{\left(\frac{∥u_{i}-u_{j}∥}{∥p_{i}-p_{j}∥+\epsilon }-1\right)}^{2},$$

This loss effectively suppresses distortion in triangle sizes caused by the mapping process, thereby improving the overall quality and uniformity of UV unfolding.

#### 2.3.5. Smoothness Loss

To encourage spatial smoothness and continuity in the UV mapping and to reduce local oscillations, we introduce a smoothness loss $L_{\mathrm{smooth}}$ defined as:

(14) $$L_{\mathrm{smooth}}=\frac{1}{N}\sum_{\left(i,j\right)\in N_{k}}{∥u_{i}-u_{j}∥}^{2},$$

where $N_{k}$ denotes the set of *k*-nearest neighbors. This term promotes consistent UV coordinates among spatially close points, ensuring local regularity in the parameterization.

#### 2.3.6. Cluster Loss

Cluster loss $L_{\mathrm{cluster}}$ enhances the compactness of points belonging to the same semantic category in the UV space, while encouraging separation between different categories:

(15) $$\begin{array}{cc} L_{\mathrm{cluster}}=\;\; & \frac{1}{C}\sum_{c=1}^{C}\frac{1}{|P_{c}|}\sum_{i\in P_{c}}∥u_{i}-\mu_{c}∥\\ \; & +\sum_{c_{1}\neq c_{2}}\max\left(0,m-∥\mu_{c_{1}}-\mu_{c_{2}}∥\right), \end{array}$$

where $\mu_{c}$ denotes the centroid of class *c*, and *m* is the minimum separation margin.

#### 2.3.7. Connectivity Loss

Connectivity loss $L_{\mathrm{connectivity}}$ encourages preservation of the neighborhood relationships from 3D space in the UV domain:

(16) $$L_{\mathrm{connectivity}}=\sum_{i=1}^{N}\sum_{j\in N_{k}\left(i\right)}∥u_{i}-u_{j}∥.$$

#### 2.3.8. Total Loss

The overall objective is defined as a weighted sum of multiple loss terms:

(17) $$L_{\mathrm{total}}=\sum_{i=1}^{7}\lambda_{i}L_{i}+I_{\mathrm{cyl}}\cdot \lambda_{\mathrm{cyl}}L_{\mathrm{cylindrical}},$$

where the seven sub-losses correspond to: Reconstruction Loss, Conformal Loss, Stretch Loss, Smoothness Loss, Boundary Loss, Cluster Loss, and Connectivity Loss. The indicator function $I_{\mathrm{cyl}}$ activates the cylindrical-aware loss only when the input point cloud is identified as having a cylindrical structure.

For all experiments conducted on the *ShapeNet* dataset, the weighting coefficients are empirically set to balance the contributions of each sub-objective as follows: $\lambda_{\mathrm{recon}}=0.3$, $\lambda_{\mathrm{conf}}=0.8$, $\lambda_{\mathrm{stretch}}=0.5$, $\lambda_{\mathrm{smooth}}=0.5$, $\lambda_{\mathrm{boundary}}=1.0$, $\lambda_{\mathrm{cluster}}=0.8$, $\lambda_{\mathrm{connect}}=1.0$, and $\lambda_{\mathrm{cyl}}=1.2$. These values were determined empirically to ensure stable convergence and balanced gradient magnitudes across all loss components.

### 2.4. Dataset

All experimental data are drawn from the PartNet [28] subset of the ShapeNet dataset. PartNet [28] is a large-scale 3D part segmentation dataset designed to support fine-grained semantic understanding and part-level analysis. Based on ShapeNet CAD models, it covers 24 object categories (e.g., chairs, tables, and knives), comprising over 26,000 models with more than 573,000 annotated parts. Each model provides hierarchical part-level semantic annotations, enabling multi-level segmentation from the whole object down to fine-grained sub-parts. For example, a chair can be decomposed into the seat, backrest, armrests, and legs, while each leg can be further divided into the front leg, back leg, and footpad. Additionally, the dataset provides 3D meshes and point clouds sampled from the meshes, where each vertex or point is associated with a corresponding part label. PartNet [28] supports a wide range of research tasks, including part segmentation, part recognition, hierarchical structure analysis, and 3D part synthesis and reconstruction.

For the generalization experiments, we selected several samples from the ShapeNetCore [29], 3D-FUTURE [30], and ModelNet [31] datasets for testing.

### 2.5. Metric

We propose a comprehensive multi-dimensional evaluation metric system to assess the quality of UV parameterization from three key aspects: geometric fidelity, structural consistency, and computational efficiency.

For geometric fidelity, we adopt a conformality metric to measure angular distortion during the parameterization process and use the Chamfer distance to quantify the geometric accuracy of the reconstructed surface. For structural consistency, we design a boundary compactness metric to evaluate the alignment accuracy between UV seams and semantic boundaries. Furthermore, we used several seam-related metrics to report seam fragmentation and complexity, including UV seams, island count, and total seam length (TSL). These metrics directly indicate seam complexity and layout compactness. For texture quality, we introduce two metrics. The texture distortion metric measures the degree of texture stretching or compression caused by the UV mapping process, reflecting the difference between the projected texture and the ideal texture mapping. We also introduce the Mean Texture Error (MTE), which computes the average difference between the target and actual texture coordinates across the entire surface, providing a reliable evaluation of texture consistency.

These metrics collectively provide a succinct and effective way to evaluate the quality of UV parameterization, encompassing geometry preservation, seam-aware structural coherence, and texture fidelity.

## 3. Results

All experiments are conducted on individual objects selected from the PartNet [28] subset of the ShapeNet dataset [28,29].

We compare UVSegNet with recent learning-based UV parameterization methods, including Nuvo, as well as classical methods such as AtlasNet [13]. The effectiveness of the proposed framework and its individual components is further evaluated through comprehensive ablation studies. All experiments are performed on a single NVIDIA RTX 4060 Ti GPU. In the end, generalization experiments are conducted on the ShapeNetCore [29], 3D-FUTURE [30], and ModelNet [31] datasets.

### 3.1. Comparative Experiment

This work conducted extensive experiments on the ShapeNet dataset to compare UVSegNet with other state-of-the-art methods (Nuvo) as well as classical methods (AtlasNet [13]).

#### 3.1.1. Comparison with Nuvo

**Figure 4:**
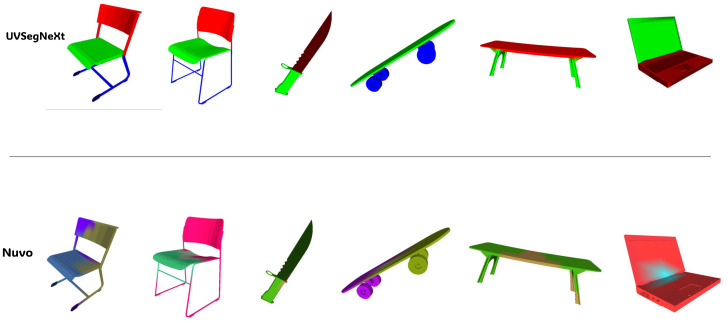
Comparison of segmentation results between Nuvo and UVSegNet. Different colors on the objects indicate different semantic parts.

Figure 4 shows the visualization of the UVSegNet mapping segmentation results, compared with Nuvo’s segmentation using probabilistic partitioning. UVSegNet outperforms Nuvo in seam alignment with semantic boundaries, topological regularity, and consistency across different object geometries.

As shown in Table 1, for the same object, UVSegNet reduces the angular distortion error to 0.1141, achieving a 24.1% improvement over the Nuvo baseline, demonstrating superior performance in preserving local geometric structures. This improvement is attributed to the semantic boundary-guided adaptive parameterization strategy, which effectively mitigates UV stretching in high-curvature regions. The proposed boundary-aware mechanism achieves a boundary compactness of 0.2360, a 60.5% increase, and successfully aligns 61.2% of UV seams with semantic boundaries. These results validate the effective fusion of semantic information and geometric features, particularly in optimizing seams at part connection regions in furniture models.

In addition, internal consistency is improved on most categories, while it is comparable or slightly worse on a small subset of objects (e.g., Chair2 and Laptop). Since internal consistency is sensitive to cross-chart smoothness, enforcing boundary-aligned charting may slightly increase this metric for a few objects, reflecting a mild trade-off between seam/structure alignment and interface smoothness. This observation is further supported by a positive correlation between boundary compactness and internal consistency across most cases (Pearsonr=0.72), suggesting that better seam alignment often coincides with more coherent parameterization. The computational efficiency of the framework has also been significantly improved, reducing the processing time from 3.7 h to just 48 min, compared to Nuvo.

In terms of texture quality, our method reduces texture distortion and ensures better preservation of texture integrity. The texture mapping results indicate more consistent alignment between texture coordinates and the 3D surface, with fewer distortions and better continuity across seams. These improvements in texture fidelity, along with the enhanced geometric preservation, demonstrate the overall effectiveness of UVSegNet in simultaneously optimizing both geometry and texture.

#### 3.1.2. Comparison with AtlasNet

Since AtlasNet [13] performs parameterization and reconstruction on the entire point cloud of a single object, we use Chamfer Distance as the main metric to measure the accuracy of geometric reconstruction. As shown in Table 2, our method outperforms AtlasNet [13] on the example objects. The conformality error is reduced by an average of 84.5% (up to 87.9%), and the Chamfer Distance decreases by an average of 70.1%. Our part-level parameterization and boundary-aware mechanism effectively preserve local geometric shapes while minimizing distortion. The overall performance trend shows that the proposed framework achieves a better balance between geometric fidelity and texture mapping accuracy. It is important to note that since AtlasNet [13] does not perform component-level segmentation, boundary compactness metrics that rely on semantic component boundaries cannot be directly applied. To provide a seam-related validity test applicable to both methods, we report the total seam length, the normalized 3D length of UV chart boundaries, which serves as a direct proxy for seam complexity (shorter is preferable for compact layouts). As shown in Table 2, UVSegNet yields consistently shorter seam boundaries than AtlasNet across all categories, indicating a more compact and less fragmented seam layout. We also observe that internal consistency is not uniformly improved across all categories, which is consistent with the trade-off between boundary-aligned chart construction and cross-chart smoothness discussed above. The consistent improvements across different object categories highlight the generalization ability of our approach and its potential for robust UV mapping in downstream tasks such as texture transfer and high-quality mesh reconstruction.

In addition to geometric quality, we also evaluate the texture quality of UV parameterization. As shown in the last two rows of the table, our method reduces texture distortion and mean texture error across all object categories. For example, the texture distortion for the chair object is reduced by 51%, indicating that our method preserves texture integrity more effectively. Similarly, the mean texture error for the laptop object is reduced by 56.4%, further emphasizing the accuracy of texture mapping. These experimental results demonstrate that our method not only improves geometric fidelity but also enhances texture mapping quality, ensuring both geometric and texture consistency.

### 3.2. Ablation Studies

As shown in Table 3, the introduction of conformal loss reduces local angular distortion during the parameterization process, highlighting its importance in preserving geometric fidelity. At the same time, removing the stretch constraint leads to a significant increase in conformal error. This coupling is expected because the stretch loss limits local scale/area distortion (texel density), and without the stretching constraint, the mapping may also suffer from severe texture stretching. Furthermore, removing the boundary loss or boundary information also affects both angular distortion and boundary compactness, reflecting their contributions to seam layout optimization. Notably, the absence of reconstruction loss significantly weakens boundary compactness (a decrease of 36.4%), suggesting that the geometric fitting process, through constraint coupling, indirectly promotes a more compact and structured boundary.

**Figure 5:**
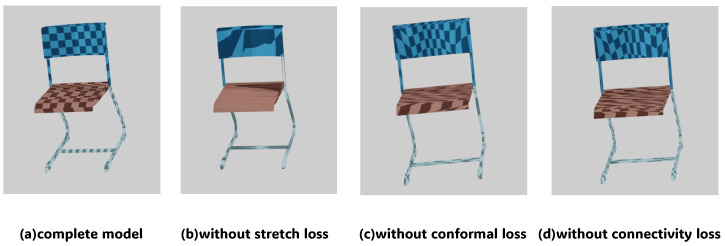
Visual comparison of ablation experiments on the chair object.

Additionally, Figure 5 shows the ablation results of the overall chair texture visualization. The full model generates smooth and continuous texture distribution, while removing the stretch loss leads to uneven local stretching and irregular checkerboard scaling. In contrast, removing the conformal loss causes angular distortion, with severe texture deformation observed in the leg region. These comparisons clearly demonstrate that both losses play a crucial role in maintaining global consistency and preserving local geometric fidelity.

### 3.3. Seam Studies

**Figure 6:**
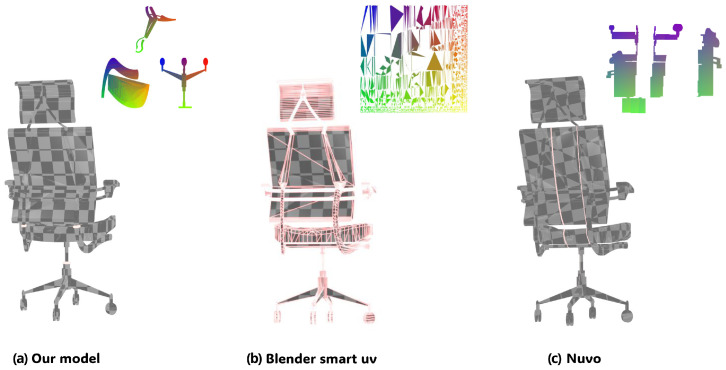
UV Seams and UV Maps of different methods. The different colors are the standard visualization of UV maps by mapping 2D texture coordinates (u, v) to colors.

Figure 6 provides a qualitative comparison of UV seam distributions generated by different methods, where (a) denotes UVSegNet, (b) Blender Smart UV, and (c) Nuvo. As shown in Figure 6a, our method produces a compact and structurally consistent seam layout, where seams are primarily aligned with meaningful part boundaries such as joints and component interfaces. This results in large, continuous UV islands and maintains texture continuity across adjacent parts. In contrast, Blender Smart UV (Figure 6b) introduces a large number of fragmented seams driven by local angle heuristics, leading to over-segmentation and disruption of texture flow.

Figure 6 provides a qualitative comparison of UV seam distributions generated by different methods, where (a) denotes UVSegNet, (b) Blender Smart UV, and (c) Nuvo. As shown in the complete model section of Figure reffig:seam, our method produces a compact and structurally consistent seam layout, where seams are primarily aligned with meaningful part boundaries, such as joints and component interfaces. This results in large, continuous UV islands and maintains texture continuity across adjacent parts. In contrast, Blender Smart UV introduces a large number of fragmented seams driven by local angle heuristics, leading to over-segmentation and disruption of texture flow. Nuvo reduces seam fragmentation to some extent but still produces redundant seams within single components, revealing its limitations in structural awareness. The quantitative results in Table 4 further support these observations. Blender Smart UV generates significantly more seams and islands, indicating over-segmentation and poor layout compactness. In contrast, Nuvo and our method only produce 3 seams and 3 islands, indicating a more compact chart segmentation. However, our method achieves a higher boundary alignment score, suggesting that the reduction in seams is achieved by aligning them with semantically meaningful component boundaries, rather than cutting within components. Overall, our method achieves seam placement more aligned with the actual structure of the object, effectively balancing seam compactness and geometric consistency, making it more practical for technical artists.

### 3.4. Qualitative Results and Generalization

**Figure 7:**
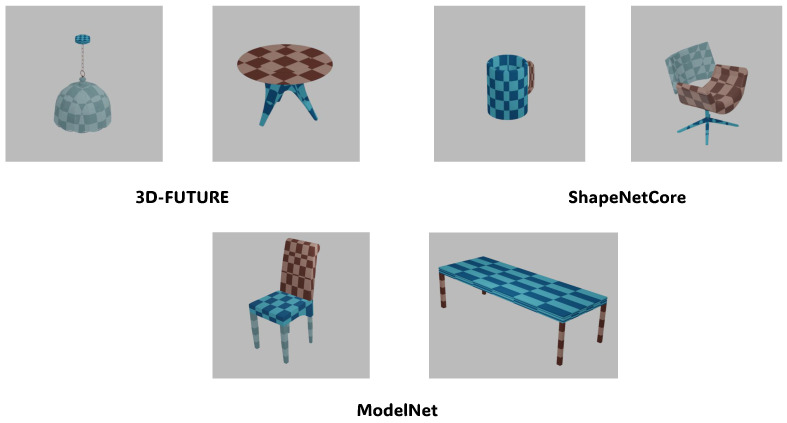
Texture mapping results of our method on other datasets. Different colors on the objects indicate different semantic parts.

**Figure 8:**
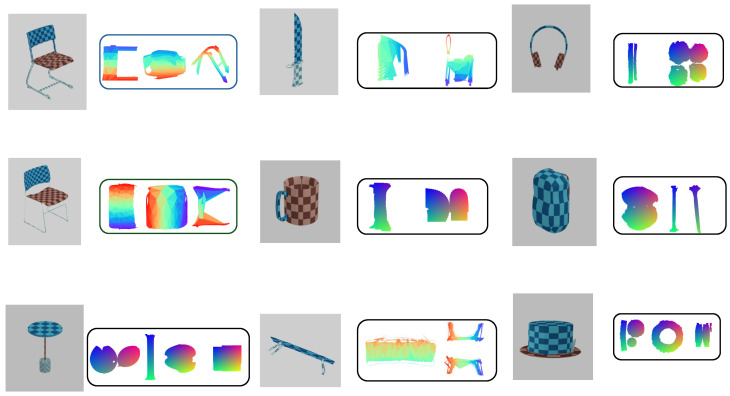
Visualization of UVSegNet outputs on various categories. Different colors on the chair indicate different semantic parts.

As shown in Figure 7, the texture mapping results of our method on three different datasets demonstrate its strong performance on the extracted samples. Figure 8 shows the visualization of UVSegNet outputs on different categories in the PartNet [28] test set, illustrating how our method effectively maps textures onto a wide range of object types, improving texture continuity and geometric accuracy.

Although our method was trained on categories from the PartNet [28] dataset, its generalization capability still performs well on object categories common to the PartNet [28] dataset, generating accurate and consistent texture mapping results. However, it is important to note that the performance of our method may decrease for categories not included in the training set. This is because our method heavily relies on semantic segmentation, and without appropriate segmentation guidance for unseen categories, the texture mapping results may become suboptimal. To improve applicability to a broader range of objects, additional semantic segmentation training or fine-tuning on more object categories may be necessary.

Qualitatively, we observe that semantic boundary awareness helps produce more structured seam layouts and improves texture continuity on many categories, consistent with the quantitative trends reported earlier. At the same time, these visual results also suggest that the overall behavior of the framework is influenced by both segmentation reliability and chart construction, motivating a further discussion of trade-offs, limitations, and generalization challenges.

## 4. Discussion

The comparative and qualitative results suggest that UVSegNet improves the overall distortion–seam trade-off by explicitly coupling semantic boundary cues with geometry-aware parameterization. Compared with purely geometry-driven or unsupervised charting baselines such as Nuvo [18] and AtlasNet [13], our framework more consistently promotes structure-aligned seam layouts and yields better texture continuity. This observation is in line with prior findings that seam placement and chart partitioning play a critical role in practical texture mapping quality beyond geometric distortion alone [23,24,32].

We also observe that the internal consistency metric is not consistently improved across all classes; on a small subset of objects, its performance is slightly inferior to competing methods. This is expected, as internal consistency is highly sensitive to cross-graph smoothness at graph interfaces, and UVSegNet explicitly encourages boundary-aligned graph drawing based on part semantics. In multi-graph parameterization, stronger cuts or graph constraints can introduce sharper graph boundaries and affect interface regularity, reflecting a common trade-off between seam decisions and smoothness [16,33,34]. In our experiments, the magnitude of the difference remains limited, suggesting that UVSegNet generally maintains mapping consistency, prioritizing structurally consistent seam placement.

Generalization results further show that the framework performs best on categories whose part semantics are well covered by the PartNet training distribution [28]. When applied to out-of-distribution categories, the reliance on part-level segmentation priors can lead to less reliable boundary cues, which propagates to seam graph construction and chart optimization. In particular, shapes with extreme topologies that are rare or underrepresented in PartNet—such as high-genus objects with multiple holes/handles, very thin or near-self-contacting structures (e.g., thin shells, wires, closely adjacent parts), and non-manifold or noisy geometries (e.g., imperfect reconstructions)—tend to produce ambiguous local neighborhoods and unstable boundary evidence, making part segmentation and boundary-driven seam guidance more challenging. This limitation is consistent with the general sensitivity of segmentation-driven pipelines to dataset bias and topology irregularities in point-based shape understanding [25,26].

Overall, these results highlight that semantic boundary guidance provides clear benefits for seam plausibility and texture continuity, while the remaining failure cases are largely tied to segmentation reliability and interface smoothness at chart boundaries. This prompts us to pursue future improvements in two complementary directions: enhancing segmentation robustness under domain shift (e.g., broader training coverage or adaptation) and introducing lightweight cross-chart consistency regularization to improve interface smoothness without sacrificing boundary-aligned seam placement.

## 5. Conclusions

This paper presents UVSegNet, a semantic boundary-aware neural UV parameterization framework that integrates part-level point-cloud segmentation with geometry-aware mapping and seam optimization. By jointly leveraging semantic boundary cues and geometric regularization (conformal and stretch constraints), UVSegNet produces UV layouts with lower distortion, more structure-consistent seam placement, and improved texture fidelity compared with strong baselines such as Nuvo and AtlasNet. In addition, the proposed cylindrical-structure-aware supervision further stabilizes unfolding for tubular parts, improving texture continuity in challenging regions. Beyond performance improvements, UVSegNet also demonstrates several key properties and potential values:

- Geometry-semantic consistency:Seam distributions are well aligned with functional part boundaries, naturally supporting downstream semantic editing and localized texture replacement.
- Structure awareness: Through cylindrical structure detection and the proposed Cylindrical Loss, the framework significantly improves the unfolding quality of tubular parts, mitigating common distortions and seam breakages.
- Scalability: With its modular two-stage design (segmentation and parameterization), UVSegNet can adapt to different resolutions and diverse 3D object categories, demonstrating strong generalization capability.

Despite these promising results, UVSegNet remains dependent on segmentation priors and can be challenged by out-of-distribution categories and extreme topologies (e.g., high-genus shapes, very thin or near-self-contacting structures, and non-manifold/noisy geometries).

Future work will focus on improving robustness to domain offsets by expanding segmentation coverage and exploring domain adaptation, and introducing lightweight cross-graph consistency regularization to better balance seam location and interface smoothness in boundary alignment. Furthermore, we plan to explore domain adaptation techniques to mitigate the impact of dataset bias and investigate joint learning strategies to decouple segmentation from parameters, thereby enhancing the framework’s generalization ability and robustness across a wider range of object categories and more complex scenes. This will contribute to the more automated and reliable construction of high-quality 3D assets.

## Figures and Tables

**Table 1:** Comparison of UV parameterization metrics across multiple object categories. Arrows indicate the optimization direction (↓ lower is better, ↑ higher is better).

Metric	Chair		Chair2		Laptop	
	Nuvo	**Ours**	Nuvo	**Ours**	Nuvo	**Ours**
Conformality ↓	0.1504	**0.1141**	0.1427	**0.0719**	0.2257	**0.0762**
Internal Consistency ↓	0.1394	**0.0188**	**0.0032**	0.0146	**0.1242**	0.3455
Boundary Compactness ↑	0.1470	**0.2360**	0.1305	**0.1010**	0.1627	**0.1794**
Texture Distortion ↓	0.2153	**0.1054**	0.1876	**0.0893**	0.3128	**0.0897**
Mean Texture Error ↓	0.1425	**0.0621**	0.1234	**0.0549**	0.2456	**0.0784**
Metric	Table		Knife		Skateboard	
	Nuvo	**Ours**	Nuvo	**Ours**	Nuvo	**Ours**
Conformality ↓	0.2088	**0.1742**	0.1956	**0.1220**	0.1597	**0.0942**
Internal Consistency ↓	0.0985	**0.0022**	0.0737	**0.0014**	0.1137	**0.0093**
Boundary Compactness ↑	0.2620	**0.2858**	0.2681	**0.3214**	0.0523	**0.1636**
Texture Distortion ↓	0.2554	**0.1652**	0.3127	**0.2021**	0.2971	**0.1718**
Mean Texture Error ↓	0.1832	**0.1143**	0.2012	**0.1370**	0.1825	**0.0953**

Note: Best results are highlighted in bold.

**Table 2:** Comparison of our method with AtlasNet across six object categories. Arrows indicate the optimization direction (↓ lower is better, ↑ higher is better).

Metric	Chair		Chair2		Laptop	
	AtlasNet	**Ours**	AtlasNet	**Ours**	AtlasNet	**Ours**
Conformality ↓	0.4721	**0.1141**	0.5921	**0.0719**	0.6194	**0.0762**
Internal Consistency ↓	**0.0024**	0.0188	0.1338	**0.0146**	**0.0031**	0.3455
Chamfer Distance ↓	0.1609	**0.0887**	0.0905	**0.0234**	0.2968	**0.0262**
Total Seam Length ↓	2.5791	**2.3324**	2.6313	**2.4133**	2.4449	**1.7322**
Texture Distortion ↓	0.2153	**0.1054**	0.1876	**0.0893**	0.3128	**0.0897**
Mean Texture Error ↓	0.1425	**0.0621**	0.1234	**0.0549**	0.2456	**0.0784**
Metric	Table		Knife		Skateboard	
	AtlasNet	**Ours**	AtlasNet	**Ours**	AtlasNet	**Ours**
Conformality ↓	0.7503	**0.1742**	0.8119	**0.1220**	0.5020	**0.0942**
Internal Consistency ↓	0.0037	**0.0022**	0.0023	**0.0014**	**0.0083**	0.0093
Chamfer Distance ↓	0.4098	**0.0079**	0.4821	**0.0089**	0.5389	**0.0136**
Total Seam Length ↓	2.4598	**1.6890**	2.3037	**1.4032**	2.5517	**1.8093**
Texture Distortion ↓	0.2554	**0.1652**	0.3127	**0.2021**	0.2971	**0.1718**
Mean Texture Error ↓	0.1832	**0.1143**	0.2012	**0.1370**	0.1825	**0.0953**

Note: Best results are highlighted in bold.

**Table 3:** Ablation study on the impact of each component. Arrows indicate the optimization direction (↓ lower is better, ↑ higher is better).

Experimental Setting	Conformality ↓	Boundary Compactness ↑
complete model	0.1141	0.2360
without boundary edge	0.1255	–
without conformal loss	0.1811	0.2061
without stretch	0.2872	0.0186
without boundary	0.1446	0.2177
without reconstruction	0.1288	0.1502

**Table 4:** Qualitative comparison of UV seam quality. Arrows indicate the optimization direction (↓ lower is better, ↑ higher is better).

Method	UV Seam Count ↓	UV Island Count ↓	Boundary Alignment Score ↑
Blender Smart UV	197	78	0.21
Nuvo	3	3	0.45
Ours (UVSegNet)	**3**	**3**	**0.93**

Note: Best results are highlighted in bold.

## Data Availability

The data presented in this study are available in Hugging Face repository, ShapeNet/PartNet-archive at https://huggingface.co/datasets/ShapeNet/PartNet-archive/tree/main (accessed on 30 May 2025), Princeton ModelNet repository at https://modelnet.cs.princeton.edu/ (accessed on 27 December 2025), Alibaba Tianchi dataset repository at https://tianchi.aliyun.com/dataset/98063 (accessed on 27 December 2025).
